# Direct comparison of performance of single nucleotide variant calling in human genome with alignment-based and assembly-based approaches

**DOI:** 10.1038/s41598-017-10826-9

**Published:** 2017-09-08

**Authors:** Leihong Wu, Gokhan Yavas, Huixiao Hong, Weida Tong, Wenming Xiao

**Affiliations:** National Center for Toxicological Research, US Food and Drug Administration, 3900 NCTR RD, Jefferson, AR 72079 USA

## Abstract

Complementary to reference-based variant detection, recent studies revealed that many novel variants could be detected with *de novo* assembled genomes. To evaluate the effect of reads coverage and the accuracy of assembly-based variant calling, we simulated short reads containing more than 3 million of single nucleotide variants (SNVs) from the whole human genome and compared the efficiency of SNV calling between the assembly-based and alignment-based calling approaches. We assessed the quality of the assembled contig and found that a minimum of 30X coverage of short reads was needed to ensure reliable SNV calling and to generate assembled contigs with a good coverage of genome and genes. In addition, we observed that the assembly-based approach had a much lower recall rate and precision comparing to the alignment-based approach that would recover 99% of imputed SNVs. We observed similar results with experimental reads for NA24385, an individual whose germline variants were well characterized. Although there are additional values for SNVs detection, the assembly-based approach would have great risk of false discovery of novel SNVs. Further improvement of *de novo* assembly algorithms are needed in order to warrant a good completeness of genome with haplotype resolved and high fidelity of assembled sequences.

## Introduction

Detection of genetic variants such as SNVs, insertions and deletions (INDELs), and structural variants (SVs) is one of the major objectives for the usage of next generation sequencing (NGS) in human genome research. Currently, genetic variant calling is based on alignment of raw sequence reads against a reference genome. This alignment-based approach has many limitations including incompleteness of genome assembly^1^, structural variations existing in the genomes of normal individuals^2^, sequencing errors in reads, and interference of single nucleotide polymorphisms (SNP) on reads mapping^3^. Thus, high levels of false positives of variant calling are reported for the alignment-based approach. In bacteria and other organisms with a small size of genome, read sequences can be assembled into long contigs, and subsequent variants can be identified via an assembly-based approach.

Although the *de novo* assembly-based approach has been considered as the ideal for genetic variants detection^4, 5^, it has not been widely applied on large and complex genomes. Recently, several attempts using this approach for human subjects have been reported^6–8^. Hundreds of thousands of novel mutations were identified in *de novo* assembled personal genomes^8^. However, there is no direct comparison with alignment-based calling to demonstrate the reliability of assembly-based variant calling. It is of interest to see if SOAPdenovo, a popular genome assembly method that was used in previous assembly-based studies^9–11^, could be suitable for the purpose of SNV calling, and to its further extension, whether coverage of reads would have some impact on the outcomes of genome assembly and SNV calling.

In this study, we assessed the performance of SNVs calling at various coverages of short reads with contigs generated by SOAPdenovo2^12^, the latest version of SOAPdenovo. We simulated short reads from the whole human genome for comparison between the assembly-based and alignment-based calling approaches. We assessed the quality of the assembled contig and determined that at least 30X coverage of sequencing reads were needed to obtain a reliable contig profile. By comparing SNVs called from alignment of assembled contigs and from alignment of reads to the “ground truth” (SNVs introduced into the template reference for simulation), we directly evaluated the performance of the two variant calling approaches. We repeated this analysis process with reads sets from whole genome sequencing (WGS) of NA24385, an individual whose genome was fully sequenced and analyzed by the Genome In A Bottle (GIAB) consortium. Similar results were obtained with experimental reads. We concluded that although an assembly-based approach (with SOAPdenovo2 as the assembly tool) might serve as a complimentary method for SNVs discovery, there were many false SNVs and missed calls due to sequence difference of two alleles in a diploid genome, such as the human genome.

## Results

### Study workflow

**Figure 1:**
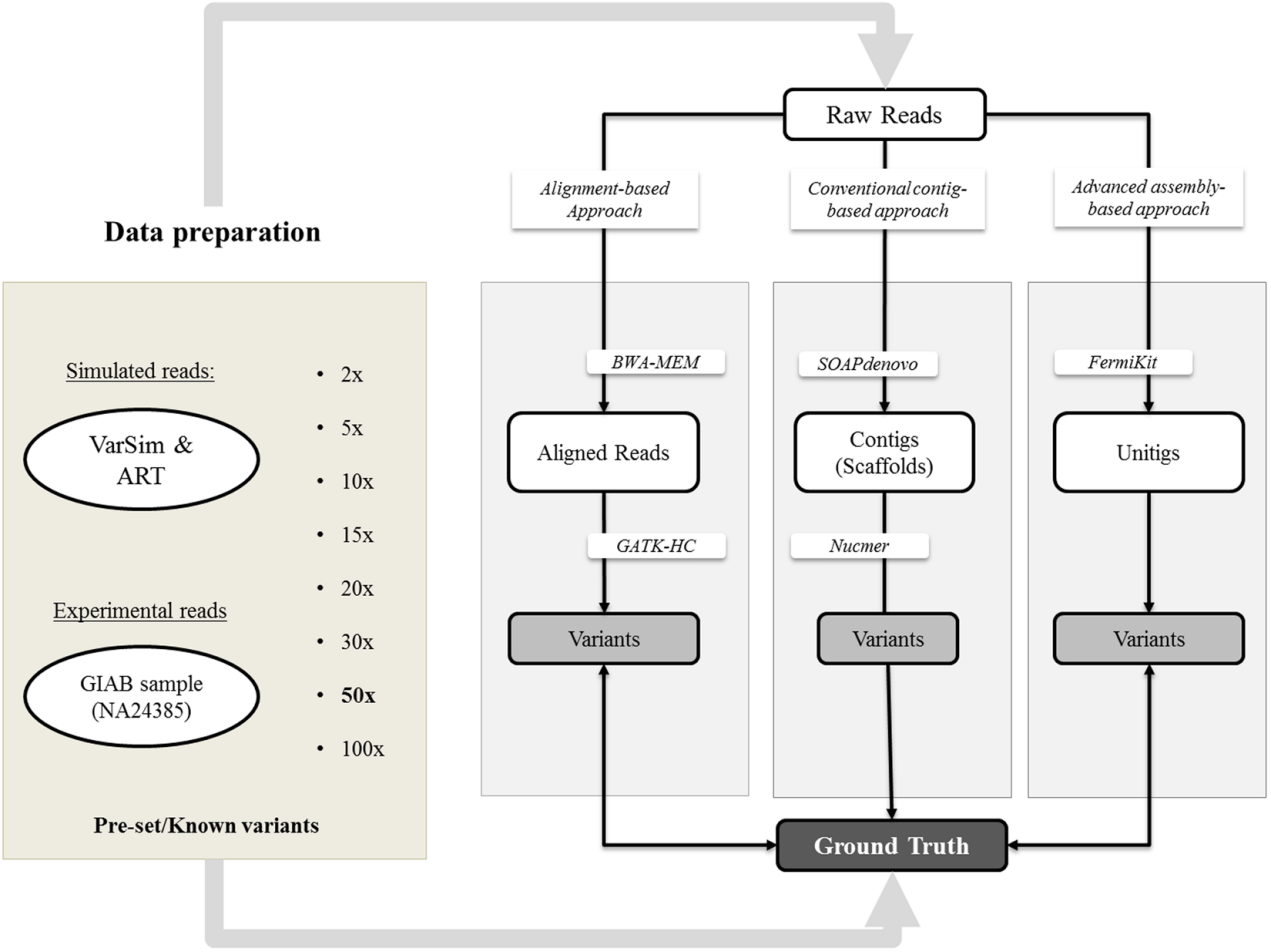
Study workflow. For data preparation, simulated reads were generated by VarSim and ART with a pre-set variant pool. Experimental reads from GIAB project (NA24385) were used for validation. Both alignment-based and assembly-based variant calling approaches were applied on these two data sets for comparison, and the variants called by different pipelines were compared to the ground truth, variants introduced into the template reference for simulation or high confident germline variants for individual NA24385.

The overall workflow is described in Fig. 1. First, ~3.6 million variants were randomly selected from a variant pool and then introduced into the human reference genome to generate the template genome for reads simulation. The simulated sets of reads were generated at coverages of 2X, 5X, 10X, 15X, 20X, 30X, 50X, and 100X. Then, both alignment-based and assembly-based calling pipelines were applied to those sets of reads for variant calling. The conventional alignment-based variant calling pipeline was implemented with a software package of BWA-MEM and GATK, whereas SOAPdenovo2 was used to generate contigs which were then mapped back to the reference genome by Nucmer. The SNV callings from the alignment of assembled contigs were done by the “show-snps” executable in the MUMmer package. In addition, we applied FermiKit to call variants based on *de novo* assembled unitigs. Recall rate and precision were then calculated for three variant calling approaches. Finally, variants from the alignment-based and assembly-based processes were compared to identify variants that were missed by the alignment-based but recovered by the assembly-based approach.

To validate conclusions derived from simulated data set, we repeated this valuation process with a date set of WGS reads for individual NA24385^13^. We used a “downsampling” approach to create eight subsets of reads from 2X to 100X coverages, as used in simulated sets, by randomly extracting reads from the original set of WGS reads (total of 300X coverages). We used high confident variant calls on this individual provided by the consortium as ground truth to evaluate the precision and rate of recall from alignment-based and assembly-based approaches.

### Quality metrics for de novo assembled contigs

From the basic statistics of assembled contigs by SOAPdenovo2 with simulated reads, we observed a dramatic increase in number of contigs and total assembly length between coverage 2X and 30X. These values were stabilized from 30X up to 100X (Table 1). Even though we did not observe further improvement in total number and length of contigs between 30X and 100X, the maximum length of contigs for 100X coverage was double that of 30X, suggesting the benefit of higher coverage of reads for further extension of assembled contigs. This process was repeated with reads set from NA24385, in which, however, we observed continuous modest increase of number of contigs and total assembly length.

**Table 1:** Statistics of *de novo* assembly result with different reads coverages.

Coverage	Dataset	# Contigs	Total contig length	Max. length	N 50	Dataset	# Contigs	Total contig length	Max. length	N50
**2x**	**Simulated Data**	140,788	14,283,137	2,692	95	**Real Data (From NA24385)**	369,652	41,624,188	6,467	127
**5x**		1,162,253	18,299,215	6,908	210		2,402,265	433,500,650	42,569	222
**10x**		6,347,989	1,367,683,599	9,424	252		8,074,740	1,978,632,975	42,639	307
**15x**		9,550,045	2,507,407,976	14,375	346		9,456,270	2,778,146,700	42,633	464
**20x**		9,754,125	3,010,504,627	29,442	556		9,394,803	3,126,114,481	31,095	783
**30x**		9,941,090	3,322,715,810	32,565	1351		9,335,130	3,343,901,059	49,256	1720
**50x**		9,745,194	3,403,686,068	66,702	3090		10,064,305	3,467,308,411	65,800	2450
**100x**		8,279,679	3,316,291,539	80,998	3736		11,571,404	3,592,236,269	72,930	2362

**Figure 2:**
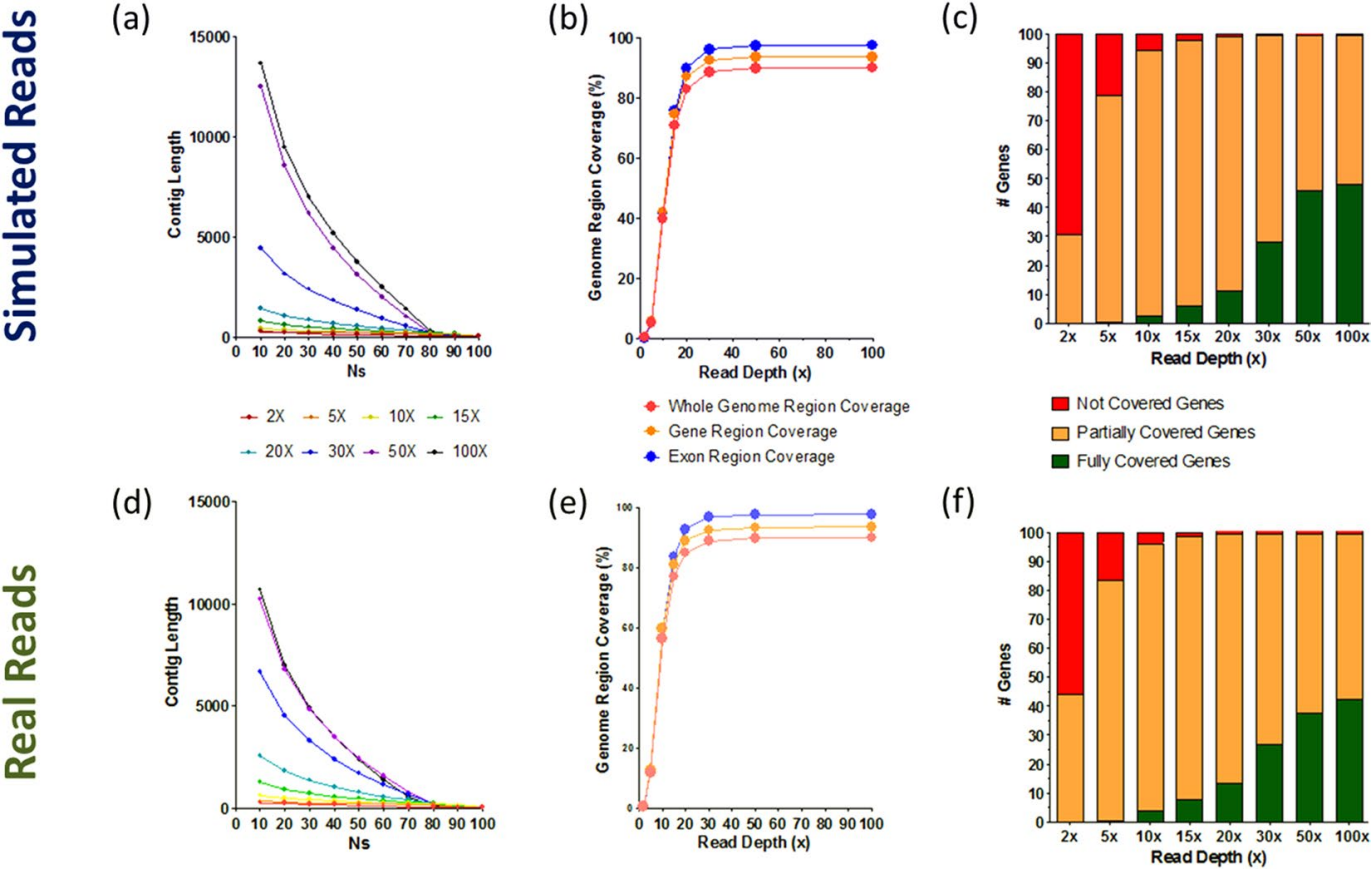
Contig continuity, genome and gene coverages for de novo assembly with SOAPdenovo2. (**a**,**d**) N statistics for different coverages. (**b**,**e**) Coverage of genome, gene, and exon regions by contigs. (**c**,**f**) Number of genes covered by assembled contigs. Fully covered gene: all regions in the gene were covered by mapped contigs. Partially covered gene: only part of regions in the gene were covered by mapped contigs. (**a–c**) Statistics of simulated reads. (**d–f**) Statistics of experimental reads.

An *Nx* plot demonstrated the *Nx* values, with ranges between 0–100%, where *Nx* is defined as the length of the shortest contig in the set of the X% largest contigs that represents at least X% of the assembly. Here, we used an *Nx* plot to present a better picture on the continuity of contigs against coverage of reads. We observed the continuous benefit in contig length when increasing coverage of reads between 2X and 50X (Fig. 2a,d). There was a large improvement on contig continuity from 30X to 50X, while this difference was not as such obvious in statistics presented in Table 1. In addition, we noticed that the continuity of contigs did not gain much when 100X reads were used.

We also investigated the coverage of genome, genes and exons by the assembled contigs against the coverage of reads used in the *de novo* assembly by aligning contigs to the reference genome. When we combined fully and partially covered genes or exons, we observed a similar pattern of increasing coverage by assembled contigs on genome, genes, and exons with the increasing coverage of reads (Fig. 2b,e). Again, all three curves were stabilized at around 80% to 90% when reads coverage reached 30X and beyond. By extending the length of the assembled contigs, the increasing coverage also improved the number of fully covered genes and exons. However, for both simulated and experimental reads data sets, further increasing of reads coverages from 50X to 100X did not see much of benefit for the coverage of genes and exons (Fig. 2c,f). These results were consistent with the results for continuity of assembled contigs (Fig. 2a,d **)**.

From the quality metrics, we demonstrated that larger coverage of reads would always result in better assembly outcomes, whereas the contig profile generated with low coverage (<=10X) was mostly incomplete for gene coverage. For 100 bp pair-end read sets with 50X coverage, almost all genes could be fully or partially covered by *de novo* assembled contigs. This statistic did not improve much at 100X coverage of reads. Therefore, for comparison of variant calling between alignment-based and assembly-based approaches, we only investigated their performance with reads coverages between 10X and 50X.

### Performance of variant calling

**Figure 3:**
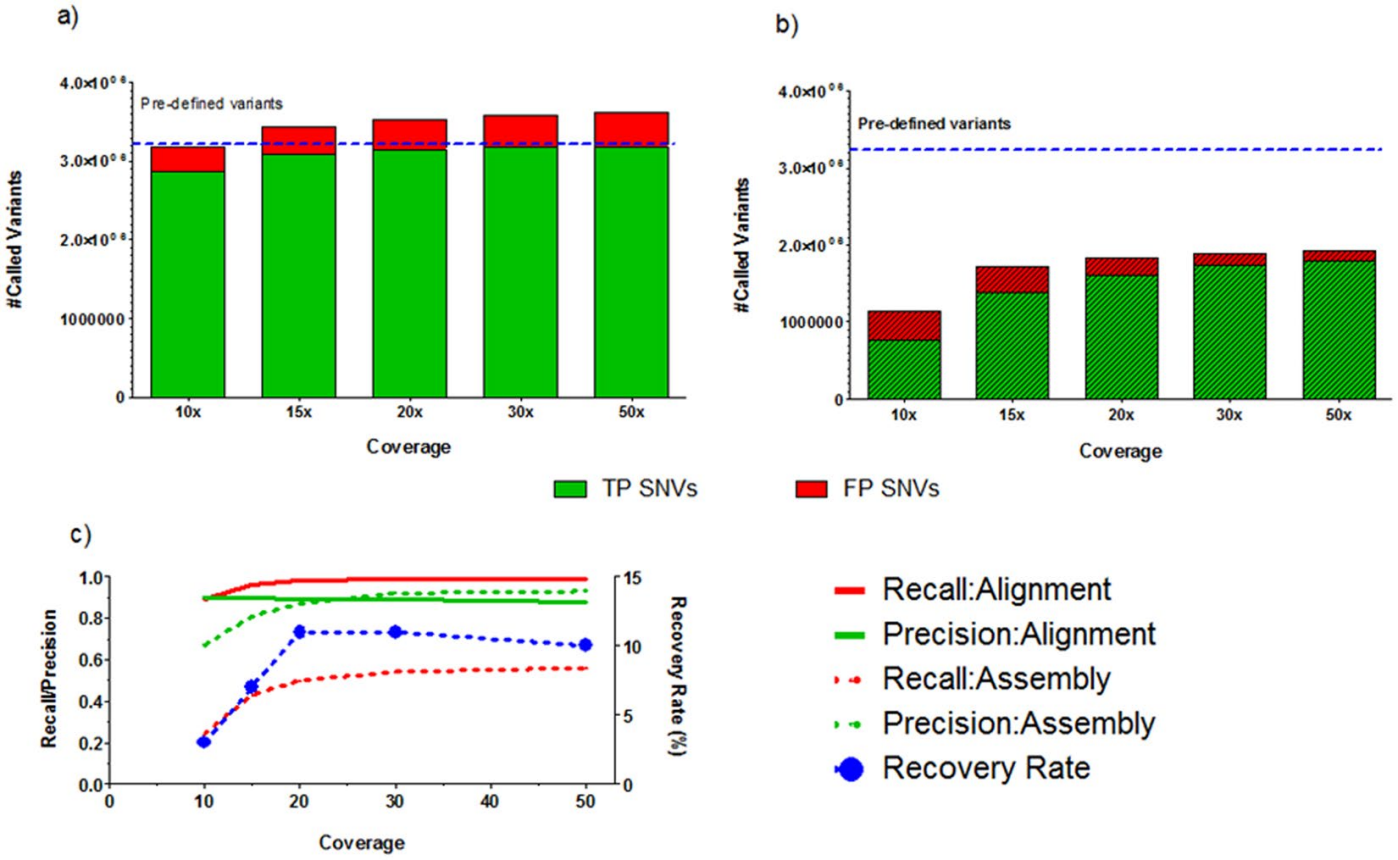
Performance of variant calling. (**a**) Alignment-based approach; (**b**) Assembly-based approach. Green and red bars represented true SNVs and false positives, respectively. (**c**) The recall and precision of both alignment-based and assembly-based variant calling. Red and green solid lines were recall rate and precision for alignment-based variant calling, respectively. Dashed red and green lines were recall rate and precision for assembly-based variant calling, respectively, whereas the dashed blue line was the recovery rate of missed variants in alignment-based variant calling.

We compared the “ground truth” with the variants called at all coverages of simulated reads from both the alignment-based and assembly-based processes to calculate the recall rates and precisions as described in the methods section. With the alignment-based variant calling, the number of true SNVs called continuously increased until 30X coverage of reads (Fig. 3a). Interestingly, the number of false SNV calls was also increased along with coverage within this span. We did not observe further increase of either true SNVs or false SNVs at higher than 30X coverage of reads, suggesting that a 30X coverage of reads is sufficient for the alignment-based approach. More than 99% of SNVs were successfully recalled with 30X coverage of reads (Table 2). For the assembly-based approach, we also observed continuous increase of true SNVs along with increase of reads coverage until it reached a plateau at 30X (Fig. 3b). It is worth noticing that the number of true SNVs and total called SNVs from the assembly-based approach were significantly lower than those from the alignment-based approach. With 50X coverage of reads, recall rate for the assembly-based approach was only 56% (Table 2). The high false negative rate might be due to low contig coverage on the whole human genome. As shown in Fig. 3c, the recall rate for the alignment-based approach reached a plateau at 30X coverage of reads, while the precision curve was pretty steady at around 90% throughout all the tested coverage range. In contrast, we observed two parallel curves of recall rate and precision with the assembly-based approach. Moreover, both recall rate and precision for the assembly-based calling were significantly lower than ones for the alignment-based approach.

**Table 2:** Variant calling result from alignment-based and assembly-based approaches.

	Coverage	10x	15x	20x	30x	50x
**Alignment-based Variant Calling**	Recall	0.89	0.96	0.98	0.99	0.99
	Precision	0.90	0.90	0.89	0.89	0.88
	Missed variants	341,558	120,804	54,591	25,788	17,850
**Assembly-based Variant Calling**	Recall	0.24	0.43	0.50	0.54	0.56
	Precision	0.67	0.81	0.87	0.92	0.93
	Recovered Variants	10,843	9,060	5,756	2,892	1,797
	Recovery Rate	3%	7%	11%	11%	10%

We further investigated variants which were missed in the alignment-based variant calling process and checked how many of them were recovered by the assembly-based approach. As shown in Table 2, even with 50X coverage of reads, the alignment-based approach missed 17,850 SNVs, of which 10% were recovered by the assembly-based approach. These results suggest that the assembly-based approach could be used as a complementary method to the alignment-based approach. However, at lower coverages, the assembly-based variant calling could not recover a significant number of SNVs due to the low quality of the assembled contigs.

Finally, we explored the possible reasons why so many SNVs failed to be called with the assembly-based approach by examining allele types of the SNVs introduced in reads at all coverages. As shown in Table 3, SNVs recalled by the assembly-based approach were predominantly homozygous, whereas SNVs failed to be recalled were predominantly heterozygous. This finding indicates a systematic error in the assembly-based approach that leads to recall bias for homozygous SNVs.

**Table 3:** Ratio of two allele types within recalled and missed SNVs by assembly-based approach.

Coverage	10x	15x	20x	30x	50x
**Homo/Hetero ratio: recalled**	2.04	1.79	1.72	1.65	1.55
**Homo/Hetero ratio: missed SNV**	0.47	0.29	0.21	0.17	0.17

We also investigated the performance on INDEL discovery by both approaches. For the alignment-based approach, the precisions were constantly maintained around 80% and recall rates were gradually improved from 52 to 63% for coverage of 10X and 30X respectively. Like SNV calling, the performance of INDEL calls were indistinguishable with reads coverage of 30X and 50X (Suppl Table s1). On the other hand, the precision and recall rate for INDEL calling by the assembly-based approach were dropped to 13% and 11% respectively, indicating its insignificance for calling INDELs.

**Figure 4:**
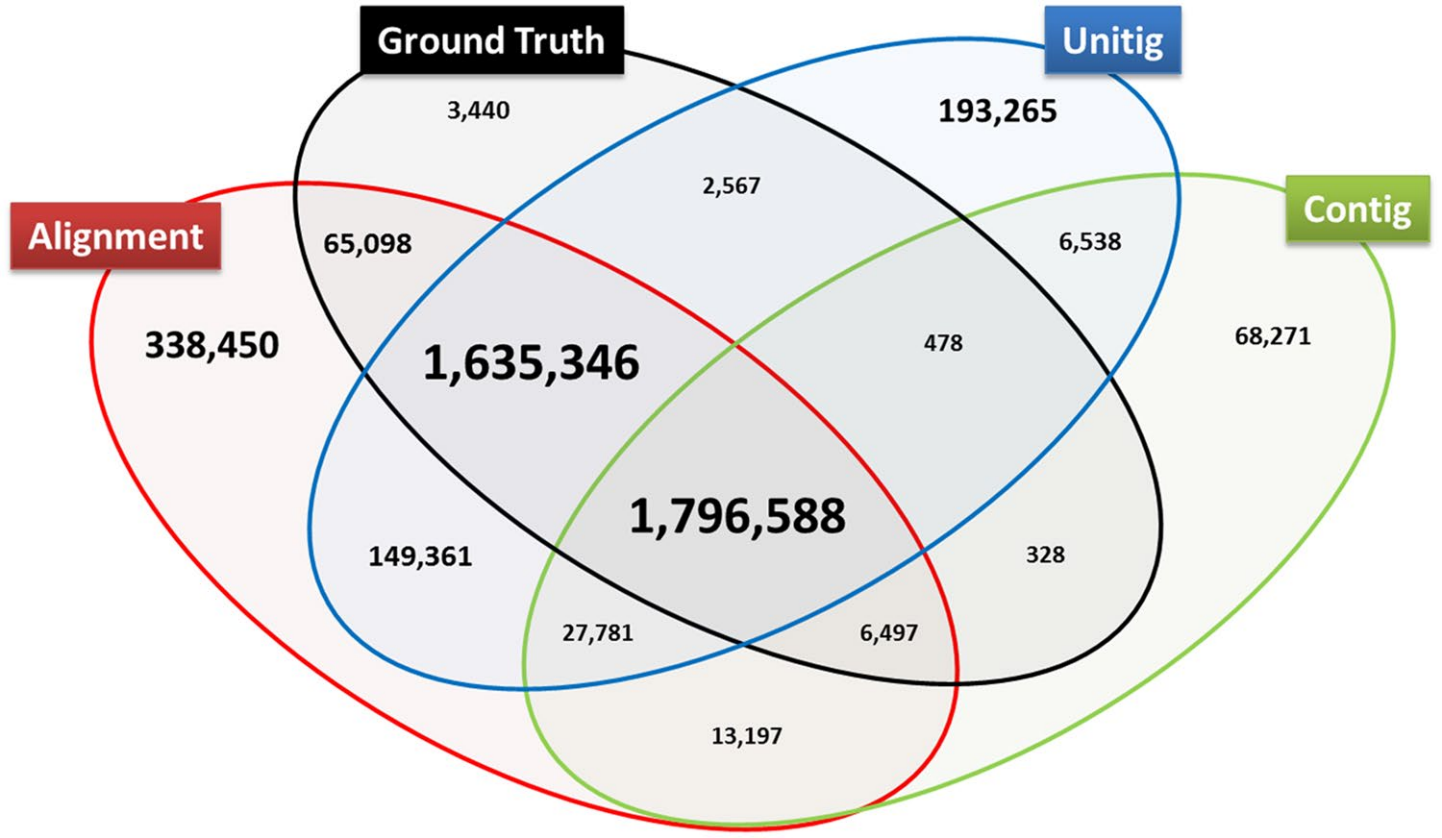
Venn Diagram to compare the performance of three variant calling algorithms to the ground truth set, retrieved from GIAB project. Alignment: variant calling with BWA-GATK pipeline; Assembly: variant calling with SOAPdenovo-MUMmer; Unitig: variant calling with FermiKit; Ground Truth: High confident calls provided by GIAB.

We repeated the evaluation process with real experiment reads for NA24385 at the coverages of 30X, 50X and 100X (Table s3). The performances of variant calling of the assembly-based approach were indistinguishable for three coverages. This result provided further evidence for the lack of need to increase short reads coverage to 100X for genome assembly with SOAPdenovo2. For experimental reads at 50X coverage (Table 4), the precision and recall rate for both alignment-based and assembly-based approaches were very comparable to the results from simulated reads (Table 3). Further assembly of contigs into scaffold did not appear to increase either on recall rate, nor precision of SNV calling. Besides MUMmer, we also tried asmVAR^14^ which used LAST^15^ as the alignment tool for contig-based variant calling. We observed higher recall rate (0.74 vs. 0.51) but lower precision rate (0.74 vs. 0.94) compared to results from MUMmer (Table 3). However, when we used FermiKit which would resolve the haplotype of contigs to uncover SNVs, its precision and recall rate were very comparable to those yielded from the alignment-based approach (Table 4). In addition, Fermikit recoverd 3,045 SNVs which accounted for 44% of SNVs missed by BWA-GATK germline SNV calling process (Fig. 4). The ability of calling INDELs by FermiKit also increased dramatically (Table s2).

**Table 4:** Variant calling performance of different approaches with 50X coverage of experimental reads.

SNV	Alignment-based approach	Assembly-based approach (SOAPdenovo)			Unitig-based approach
**Algorithm**	**BWA-GATK**	**MUMmer**		**asmVAR**	**FermiKit**
**Input Type**	**Reads**	**Contigs**	**Scaffolds**	**Contigs**	**Unitigs**
**TP**	3,503,529	1,803,891	1,810,100	2,613,576	3,434,979
**FP**	528,789	115,787	189,185	927,616	376,945
**FN**	6,813	1,706,451	1,700,242	896,766	75,363
**Recall**	0.99	0.51	0.52	0.74	0.98
**Precision**	0.87	0.94	0.91	0.74	0.88

**Figure 5:**
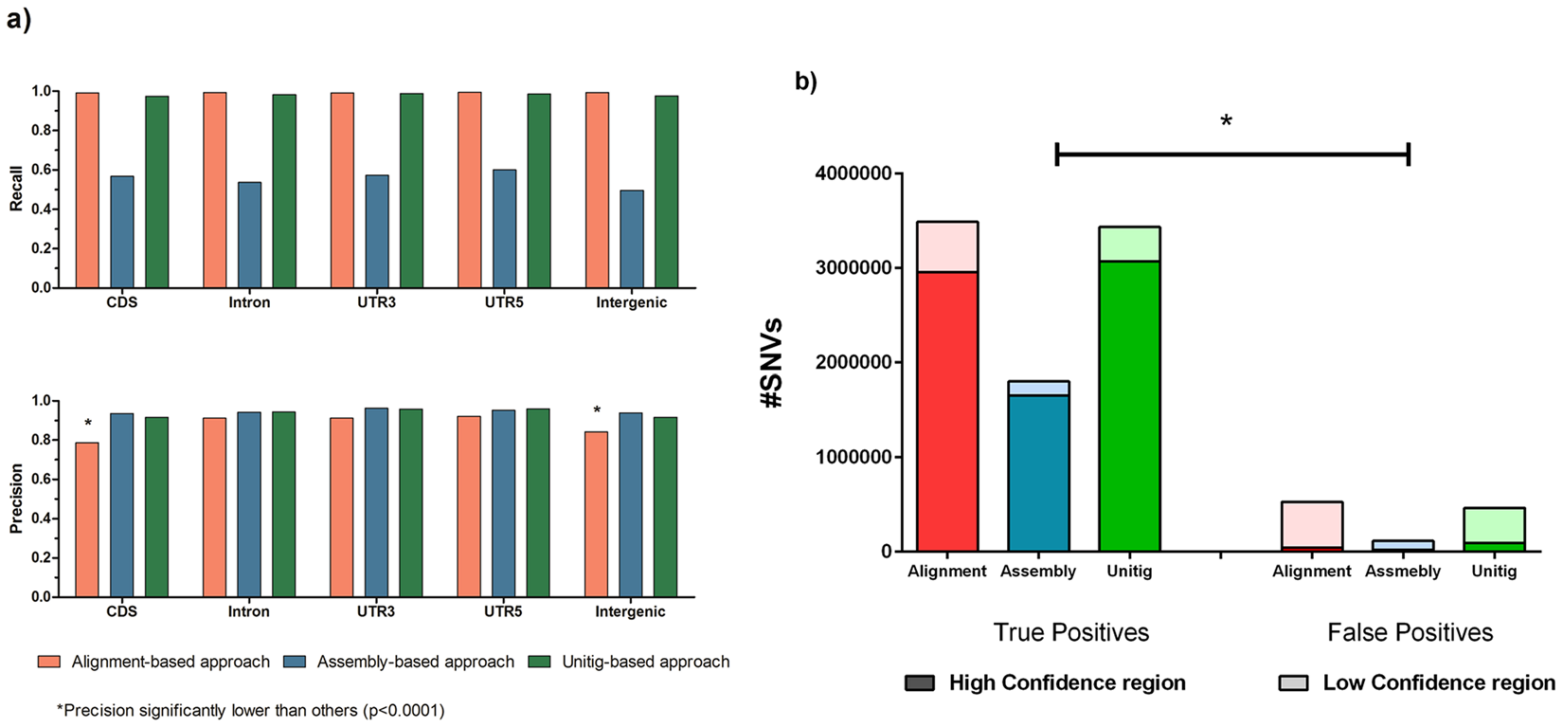
Genomic locations of variant called by three approaches. (**a**) The recall and precision ratio for each genome region. Red, blue and green bars represented alignment-based, assembly-based and unitig-based approaches, respectively. (**b**) Distribution of SNV callings in high and low confidence region. For each bar, dark color represented SNV called in high confidence region and light bar represented SNVs in low confidence region. The difference of high-confidence SNV ratio between True positive and false positive is significant (P < 0.001).

Moreover, we investigated the genomic region of variants called by each approach, which was annotated by Annovar based on refGene (http://refgene.com). As a result, we observed the variant callings on all regions (coding sequences (CDS), intronic, untranslated regions (UTRs), etc.) are evenly distributed, as they showed no significant difference in recall (Fig. 5a). We did see a lower precision in CDS and intergenic regions comparing to other genomic regions by alignment-based approach (Fig. 5a **)**. While this bias only happened to the alignment-based approach but not to the other two approaches (assembly-based and unitig-based), it suggested that the assembly-based approach would be able to partially correct such bias. Since the genome regions for NA24385 have been marked with high and low confidence based on sequence coverage by multiple sequencing platforms^16^, we sought the possible tie between confident regions and the performance of SNV calling. As we expected, regardless of which approach was used, most true positive callings were located in the high confidence regions, where false positive callings were most likely located in the low confidence regions (p < 0.001, Fig. 5b).

## Discussion

Although alignment-based variant calling is commonly used to identify genetic variants in human genomes, a high level of false positive variant calls is an issue due to multiple factors such as incompleteness of the reference genome used, a large number of SNPs and structural variants among individuals leading to mapping bias. Another approach is to use long contigs assembled from short reads to detect variants by comparison with a reference genome. The assembly-based approach has been widely used in analysis of genomes of monoploid organisms such as bacteria^17–19^. Recent studies have tried assembly-based approach on human genomes and reported hundreds of thousands of variants that lacked ground truth or supporting validation. The validity of assembly-based calling hence remains questionable. In this study, we used a random selection of ~3.6 million variants from a pool with 505 million variants to simulate short reads at various coverages. We used those sets of simulated short reads to address the following questions: (1) what is the minimum reads coverage to yield a high recall rate and precision for the alignment-based approach? (2) What is the minimum reads coverage to get good assembled contigs? (3) Will the assembly-based approach provide reliable variant calls and thus serve as a complimentary role for variant detection in human genome research?

With SOAPdenovo2, the latest version of assembler used in previous assembly-based studies^20^, we assembled the simulated short reads into long contigs. After examining several quality metrics for assembly outcome, such as continuity, contig coverage on reference genome, genes, and exons, we observed continuous benefit with increasing coverage of reads until it reached 50X, where almost all genes could be fully or partially covered by the *de novo* assembled contigs. Further increasing reads coverage to 100X did not seem increase contig continuity and coverage of genes. However, in order to get a higher coverage of genome and more fully covered genes by assembled contigs, we recommend use of a higher than 30X coverage of short reads.

We also examined the reads coverage effect on recall rate and precision of the alignment-based approach. When reads coverage was lower than 10X, we observed a large number of SNVs that were missed. However, with a 15X coverage of reads, more than 96% of SNVs were successfully recalled. When reads coverage reached 30X, 99% of the SNVs were recalled. On the other hand, the precision of SNV calling remained constant at around 90% throughout all the tested ranges, suggesting that the problem of false positives with the alignment-based approach could not be resolved by simply increasing reads coverage.

We used MUMmer for alignment of the assembled contigs against the reference genome and called SNVs with a module in the MUMmer package. MUMmer is a well-tested alignment tool for long sequence query against a large reference genome because of its high performance on speed, accuracy and scalability^21^. We therefore developed a framework with MUMmer that not only performs quality assessment for genome assembly outcomes, but also carries out assembly vs. reference alignment as the underlying driving engine and eventually makes variant calls directly.

With our framework, we were able to examine the recall rate and precision for the assembly-based variant calling process. We observed that the number of true SNVs and the total called SNVs from the assembly-based approach were significantly lower than the metrics from the alignment-based approach. With a 50X coverage of reads, recall rate for the assembly-based approach was only 56%. The curves for recall rate and precision vs. reads coverage were in parallel, suggesting that, unlike the alignment-based approach, increasing reads coverage for the assembly-based approach would have impact on both false positive and false negative SNV calls (Fig. 3c). These results were confirmed by a repeated process where experimental reads were used.

At each of the reads coverages, we examined the SNVs that were missed by the alignment-based approach to calculate the percentage of the SNVs recovered by the assembly-based process. To our surprise, even at a high coverage of short reads, only ~11% of the SNVs missed by the alignment-based approach were recovered by the assembly-based approach, suggesting that the complementary effect of the assembly-based approach was small.

Finally, we investigated the possible reasons for the low recall rate of the assembly-based approach by examining the allele types in the recalled and missed SNVs. We observed that SNVs recalled by the assembly-based approach were predominantly homozygous, whereas SNVs failed to be recalled were predominantly heterozygous, indicating a systematic error in the assembly-based approach that leads to recall bias for homozygous SNVs. The underlying algorithm used in SOAPdenovo2 is a *de Bruijn* graph that requires generation of graphic nodes with k-mer seed sequences. All possible combinations of the graphic nodes were searched within the entire input of reads. Should reads distinguish each other only due to allele differences or sequencing errors, the consensus sequence for this group of reads would be used. This error correction process would thus collapse reads from two alleles into a single haplotype. As a result, homozygous SNVs would be called correctly with the assembly-based approach, whereas heterozygous SNVs would have no more than 50% of chance to be called correctly.

Our result demonstrated that contigs generated by SOAPdenovo2 could not perform well on SNV calling for human genome, primarily due to its loss of information on read coverage and diploidy. Improvement on assembly or variant calling which overcomes current limitations might lead to better performance and make a contig-based approach more useful. As matter of fact, when we used FermiKit, an assembly tool that would preserve haplotype information in assembled contigs/unitigs, we observed precision and recall rate at very similar levels to that with alignment-based approach.

With the simulated and experimental data we evaluated the effect of reads coverage on *de novo* genome assembly with SOAPdenovo2, and SNV calling with the alignment-based and assembly-based approaches. We concluded that the higher the coverage of short reads, the better the assembly outcomes. At least 50X coverage of reads were required in order to warrant good assembled contigs that would cover 80% of the human genome. For the alignment-based SNV calling, more than 99% of SNVs could be accurately recalled at 30X coverage of reads, whereas only 56% of SNVs could be recalled by the assembly-based process at 50X coverage of reads. Nevertheless, the assembly-based process could recover merely 11% SNVs that might be missed by the alignment-based approach. The low recovery rate of SNVs by the assembly-based approach was due to inability of haplotype-resolved assembled contigs by SOAPdeno2.

## Methods

### Data simulation

We used VarSim^22^ and ART^23^ to simulate short reads with a fixed variant pool. The variants were obtained from the VarSim website as described in the quick start demo (http://web.stanford.edu/group/wonglab/varsim/), including SNVs, INDELs and SVs, primarily from dbSNP (build 144). In addition, we added an extra 400,000 SNVs from sample NA12878, reported by the Genome in a Bottle (GIAB) project (https://sites.stanford.edu/abms/giab) and thus created a pool of 505 million variants. We randomly selected ~3.6 million variants from this pool and introduced them into the human reference genome (hs37d5) with VarSim to create a template genome. We then applied this template genome to ART and simulated 100 bp pair-end reads at coverages of 2X, 5X, 10X, 15X, 20X, 30X, and 50X with introducing random errors. The fragment size of pair-end reads was set to a mean value of 350 bp with standard deviation (SD) of 50 bp.

### Whole genome sequencing reads for NA24385

Raw sequence reads for an individual, NA24385 (ftp://ftp-trace.ncbi.nlm.nih.gov/giab/ftp/data/AshkenazimTrio/HG002_NA24385_son/NIST_HiSeq_HG002_Homogeneity-10953946/HG002_HiSeq. 300x_fastq/) were downloaded from the GIAB official website. Genomic DNA of each individual was sequenced by Illumina HiSeq with 148 bp pair-ended reads at 300X coverage. Of total of 935 pair-end fastq files, there were 4 million reads in each file. We pooled the first 102, 167, and 327 files to create data sets with coverage of 30X, 50X, and 100X respectively.

In addition, germline variants in these individuals have been well characterized by the GIAB with various technology platforms and different bioinformatics discovery tools. High-confidence variant calls for these two individuals have been released by the consortium as references. We downloaded the recent release of VCF files (v3.2.2) for NA24385 (ftp://ftp-trace.ncbi.nlm.nih.gov/giab/ftp/release/AshkenazimTrio/HG002_NA24385_son/NISTv3.2.2/) and their associated BED files for high-confidence genomic regions from NCBI for this study, where high-confidence genomic regions were defined based on coverage of sequencing reads from various NGS platforms, insistency of genotype calling, and sequences homologues (https://github.com/genome-in-a-bottle/giab_FAQ).

### De novo Genome assembly

We used SOAPdenovo2 to perform *de novo* assembly with the simulated reads. Since we did not include jumping reads, pair-end reads with a large insert size, which were for the purpose of building scaffolds, we only ran the first two steps, pregraph and contig, for SOAPdenovo2 with 63-mer of seed size. We applied the same parameters for assembly processes for all coverages of the simulated reads. We used 48 CPUs on a single node of the HPC cluster with 2000 GB memory for each assembly run.

### Contig quality assessment

An in-house software package was used to assess the contig quality metrics and the coverage of the reference genome. This in-house tool, developed in Java, maximized the usage of available computational resources by performing contig alignment and post processing in parallel. Its flexible design allowed split jobs being run either on a high performance computing (HPC) cluster or a multi-core workstation. Based on carefully filtered alignment, it generated statistics such as the total genome coverage, gene and exon coverage, contig duplication as well as SNVs embedded in the assembly. This framework also provided stand-alone quality statistics such as contig size distribution, N*x* statistics, etc.

### Alignment-based and Assembly-based Variant calling

For alignment-based variant calling process we first mapped the simulated reads against the human reference genome (hs37d5, the same version used for the simulation) with BWA-MEM^24^. We then used Picard (http://broadinstitute.github.io/picard/, Version 1.110) to mark and remove repeated reads, to sort and create indexes on alignment bam files before applying the HaplotypeCaller in the GATK package (Version 3.1.1)^25^ for final variant calling.

We used MUMmer^21^ to map contigs onto the human reference genome and then called variants via Nucmer program. Nucmer (NUCleotide MUMmer) was designed for standard DNA sequence alignment and could handle multiple reference and multiple query sequences^26^. Since Nucmer could not use the whole human genome as a reference, we ran each chromosome separately and then selected the best match of each contig across all chromosomes. For instance, if one contig matched to multiple chromosomes, only the chromosome with the best matching score would be selected. We also used the same pipeline for read variant calling (BWA-MEM&GATK) to call variants from contigs, however the performance was not as good as using Nucmer.

We also used AsmVar^14^ to map contigs onto human reference genome and derived variants. To speed up the analysis process, we divided the input contigs file into 40 partitions and aligned each file separately to the reference using the lastal and last-split programs of the LAST package. More specifically, the minimum score for gapped alignments was set to 25, and the mismatch cost was set to 3 for the lastal program. For the last-split program the minimum alignment score was set to 35. The default values were used for the rest of the parameters for both programs. After the alignments were computed for each contig in separate files, the results were output in multiple alignment format (MAF) by the alignment tool. These MAF files were then merged into a single file. We then used the ASV_VariantDetector in the AsmVar package to call the SNVs for each chromosome separately with the default parameters.

FermiKit was used to call variants via a *de novo* assembly-based method^27, 28^. Different from other *de novo* assembly approaches, FermiKit assembled unitigs instead of contigs, as a lossless representation of reads^27, 28^. FermiKit used in this study was downloaded from GitHub (Sept. 2016) for the experimental reads dataset, which contained 50x of 150 bp paired-end reads. In details, the genome size was set to 3 g as human and 16 CPU cores were used in the progress.

After obtaining results from the alignment-based and assembly-based variant calling processes, we calculated recall rates and precisions for both approaches. Specifically, recall rate is the fraction of true SNVs that were called, known as TP/(TP + FN), and precision is the fraction of true SNVs among all called SNVs (TP/P), where TP (True Positive) means real positive SNVs that have been correctly called, FN (False Negative) means real positive SNVs have been wrongly called as negative, and P represents all called SNVs. In addition, we also closely examined the overlapping variants between two approaches, as well as the number of variants that were missing by the alignment-based approach but were recovered by the assembly-based approach.

Annovar^29^ was applied on genomic region analysis, to annotate the genomic region of all variants called by three different approaches respectively, based on the refGene database. Also, the variants were annotated with confidence tag, which was retrieved from the GIAB project.

### Disclaimer

The views presented in this article do not necessarily reflect current or future opinion or policy of the US Food and Drug Administration. Any mention of commercial products is for clarification and not intended as endorsement.

## Electronic supplementary material

Supplementary tables
