# Supplementary material for: Direct comparison of performance of single nucleotide variant calling in human genome with alignment-based and assembly-based approaches
Source: Sci Rep. 2017 Sep 8;7:10963. doi: 10.1038/s41598-017-10826-9 (PMC5591230; doi:10.1038/s41598-017-10826-9)
Supplement: Supplementary file 1 — Supplementary tables [file 41598_2017_10826_MOESM1_ESM.doc]

# Direct comparison of performance of single nucleotide variant calling in human genome with alignment-based and assembly-based approaches

Leihong Wu^1^, Gokhan Yavas^1^, Huixiao Hong^1^, Weida Tong^1^ and Wenming Xiao^1*^

^1^National Center for Toxicological Research, US Food and Drug Administration, 3900 NCTR RD, Jefferson, AR 72079, USA

**Table s1.** Variant calling result of INDEL with simulated data

| **INDELs** | **Coverage** | **10x** | **15x** | **20x** | **30x** | **50x** |
| --- | --- | --- | --- | --- | --- | --- |
| **Alignment-based approach** | **Total Call** | 268,358 | 305,848 | 321,557 | 334,147 | 341,263 |
|  | **TP** | 215,023 | 243,089 | 254,150 | 261,733 | 264,902 |
|  | **FP** | 53,335 | 62,759 | 67,407 | 72,414 | 76,361 |
|  | **FN** | 202,459 | 174,393 | 163,332 | 155,749 | 152,580 |
|  | **Recall** | 0.52 | 0.58 | 0.61 | 0.63 | 0.63 |
|  | **Precision** | 0.80 | 0.79 | 0.79 | 0.78 | 0.78 |
|  |  |  |  |  |  |  |
| **Contig-based approach** | **Total Call** | 229,381 | 375,120 | 432,704 | 431,610 | 356,566 |
|  | **TP** | 22,512 | 42,069 | 50,956 | 53,219 | 47,540 |
|  | **FP** | 206,869 | 333,051 | 381,748 | 378,391 | 309,026 |
|  | **FN** | 394,970 | 375,413 | 366,526 | 364,263 | 369,942 |
|  | **Recall** | 0.05 | 0.10 | 0.12 | 0.13 | 0.11 |
|  | **Precision** | 0.10 | 0.11 | 0.12 | 0.12 | 0.13 |

**Table s2.** Indel calling with experimental reads

| **INDEL** | **Alignment-based**  **approach** | **Contig-based**  **approach** | | **Unitig-based**  **approach** |
| --- | --- | --- | --- | --- |
| **Algorithm** | BWA-GATK | SOAPdenovo-MUMmer | | FermiKit |
| **Input Type** | Reads | Contigs | Scaffolds | Unitigs |
| **TP** | 633,680 | 81,039 | 86,057 | 576,077 |
| ***1bp*** | *326,764* | *81,039* | *86,057* | *310,271* |
| ***2-5bp*** | *202,454* | *0^*^* | *0^*^* | *185,692* |
| ***>5bp*** | *104,462* | *0^*^* | *0^*^* | *80,114* |
| **FP** | 255,047 | 853,081 | 1,330,496 | 185,605^**^ |
| ***1bp*** | *79,798* | *853,081* | *1,330,496* | *53,398* |
| ***2-5bp*** | *85,393* | *0^*^* | *0^*^* | *66,054* |
| ***>5bp*** | *89,856* | *0^*^* | *0^*^* | *66,153* |
| **FN** | 76,116 | 628,757 | 623,739 | 127,639 |
| **Recall** | 0.89 | 0.11 | 0.12 | 0.82 |
| **Precision** | 0.71 | 0.09 | 0.06 | 0.76 |

^*^MUMmer only called single nucleotide variants during its variant calling process (Nucmer, show-snps).

^**^Fermi may predict multiple alterations in one position, only indels with no true positive alteration were considered as false positives.

**Table s3.** Variant calling performance of contig-based approach with real data

|  | **Coverage** | **50x** | **100x** |
| --- | --- | --- | --- |
| **SNV** | Total Call | 1,919,688 | 1,873,879 |
|  | TP | 1,803,871 | 1,770,820 |
|  | FP | 115,817 | 103,059 |
|  | FN | 1,706,471 | 1,739,522 |
|  | Recall | 0.51 | 0.50 |
|  | Precision | 0.94 | 0.95 |
|  |  |  |  |
| **INDEL** | Total Call | 934,127 | 851,783 |
|  | TP | 81,038 | 74,509 |
|  | FP | 853,089 | 777,274 |
|  | FN | 628,704 | 635,233 |
|  | Recall | 0.11 | 0.10 |
|  | Precision | 0.09 | 0.09 |
